# Draft Genome Sequences of Two Ureolytic Bacteria Isolated from Concrete Block Waste

**DOI:** 10.1128/genomeA.00762-16

**Published:** 2016-08-04

**Authors:** Hongjae Park, Byeonghyeok Park, Hyun Jung Kim, Woojun Park, In-Geol Choi

**Affiliations:** a Department of Biotechnology, College of Life Sciences and Biotechnology, Korea University, Seongbuk-gu, Seoul, South Korea; b Department of Environmental Science and Ecological Engineering, College of Life Sciences and Biotechnology, Korea University, Seongbuk-gu, Seoul, South Korea

## Abstract

We sequenced genomes of two ureolytic bacteria, *Bacillus* sp. JH7 and *Sporosarcina* sp. HYO08, which were isolated from concrete waste and have a potential for biocementation applications.

## GENOME ANNOUNCEMENT

Microbiologically induced calcite precipitation (MICP) is a natural biogeochemical process in which microbial ureases often intervene (1). The biomediated production of calcite crystals has been regarded as a biocementation process that can be utilized for self-healing of concrete cracks in buildings (2). Seeking candidate microbes for biocementation, we isolated two MICP-capable bacteria, *Bacillus* sp. JH7 and *Sporosarcina* sp. HYO08, from concrete block waste collected from two deserted construction sites in South Korea (3).

The genome sequencing was carried out by the Illumina MiSeq platform using paired-end libraries with an average insert size of 500 bp. We obtained 1.46 million paired-end reads (total 851 Mbp; 152-fold coverage) and 1.94 million paired-end reads (total 1,090 Mbp; 352-fold coverage) from the JH7 and HYO08 strains, respectively. Low-quality (*Q* < 30) reads were eliminated by the HTQC program (4). The filtered reads were assembled using the SPAdes program (5). The final assembly was 5,547,983 bp with 36 scaffolds for JH7 and 3,051,793 bp with 32 scaffolds for HYO08. The *N*_50_ values for the two assemblies were 3,345,253 bp for JH7 and 413,117 bp for HYO08. Annotation of genomic sequences was performed using the NCBI Prokaryotic Genome Annotation Pipeline. A total of 5,404 and 2,891 protein-coding genes, 38 and 27 rRNA coding sequences, and 91 and 81 tRNA coding sequences for JH7 and HYO08, respectively, were identified.

### Nucleotide sequence accession numbers.

The nucleotide sequences have been deposited in GenBank under the accession numbers LPUS00000000 for *Bacillus* sp. JH7 and LPUT00000000 for *Sporosarcina* sp. HYO08.
